# Imaging-genomics reveals driving pathways of MRI derived volumetric tumor phenotype features in Glioblastoma

**DOI:** 10.1186/s12885-016-2659-5

**Published:** 2016-08-08

**Authors:** Patrick Grossmann, David A. Gutman, William D. Dunn, Chad A. Holder, Hugo J. W. L. Aerts

**Affiliations:** 1 Department of Radiation Oncology, Dana-Farber Cancer Institute, Brigham and Women’s Hospital, Harvard Medical School, Boston, MA USA; 2 Department of Biostatistics & Computational Biology, Dana-Farber Cancer Institute, Boston, MA USA; 3 Department of Neurology, Emory University School of Medicine, Atlanta, GA USA; 4 Department of Biomedical Informatics, Emory University School of Medicine, Atlanta, GA USA; 5 Department of Radiology and Imaging Sciences, Emory University School of Medicine, Atlanta, GA USA; 6 Radiology, Dana-Farber Cancer Institute, Brigham and Women’s Hospital, Harvard Medical School, Boston, MA USA

**Keywords:** Imaging-genomics, Radiomics, Glioblastoma, Volumetric, Pathways, Prediction, Noninvasive, Radiation Oncology, Neuro-imaging

## Abstract

**Background:**

Glioblastoma (GBM) tumors exhibit strong phenotypic differences that can be quantified using magnetic resonance imaging (MRI), but the underlying biological drivers of these imaging phenotypes remain largely unknown. An Imaging-Genomics analysis was performed to reveal the mechanistic associations between MRI derived quantitative volumetric tumor phenotype features and molecular pathways.

**Methods:**

One hundred fourty one patients with presurgery MRI and survival data were included in our analysis. Volumetric features were defined, including the necrotic core (NE), contrast-enhancement (CE), abnormal tumor volume assessed by post-contrast T1w (tumor bulk or TB), tumor-associated edema based on T2-FLAIR (ED), and total tumor volume (TV), as well as ratios of these tumor components. Based on gene expression where available (*n* = 91), pathway associations were assessed using a preranked gene set enrichment analysis. These results were put into context of molecular subtypes in GBM and prognostication.

**Results:**

Volumetric features were significantly associated with diverse sets of biological processes (FDR < 0.05). While NE and TB were enriched for immune response pathways and apoptosis, CE was associated with signal transduction and protein folding processes. ED was mainly enriched for homeostasis and cell cycling pathways. ED was also the strongest predictor of molecular GBM subtypes (AUC = 0.61). CE was the strongest predictor of overall survival (C-index = 0.6; Noether test, *p* = 4x10^−4^).

**Conclusion:**

GBM volumetric features extracted from MRI are significantly enriched for information about the biological state of a tumor that impacts patient outcomes. Clinical decision-support systems could exploit this information to develop personalized treatment strategies on the basis of noninvasive imaging.

**Electronic supplementary material:**

The online version of this article (doi:10.1186/s12885-016-2659-5) contains supplementary material, which is available to authorized users.

## Background

Glioblastoma (GBM) is a highly invasive and diffuse WHO grade IV tumor and is the most lethal central nervous system malignancy with an annual age-adjusted incidence rate of 3.19/100,000 per population [1]. Despite aggressive surgical therapy, radiation therapy, and temozolomide administration the 2-year survival rate remains around 27 % [2]. As a result, recent investigations have focused on capitalizing on the high molecular heterogeneity of gliomas to develop personalized treatment strategies [3].

**Fig. 1:**
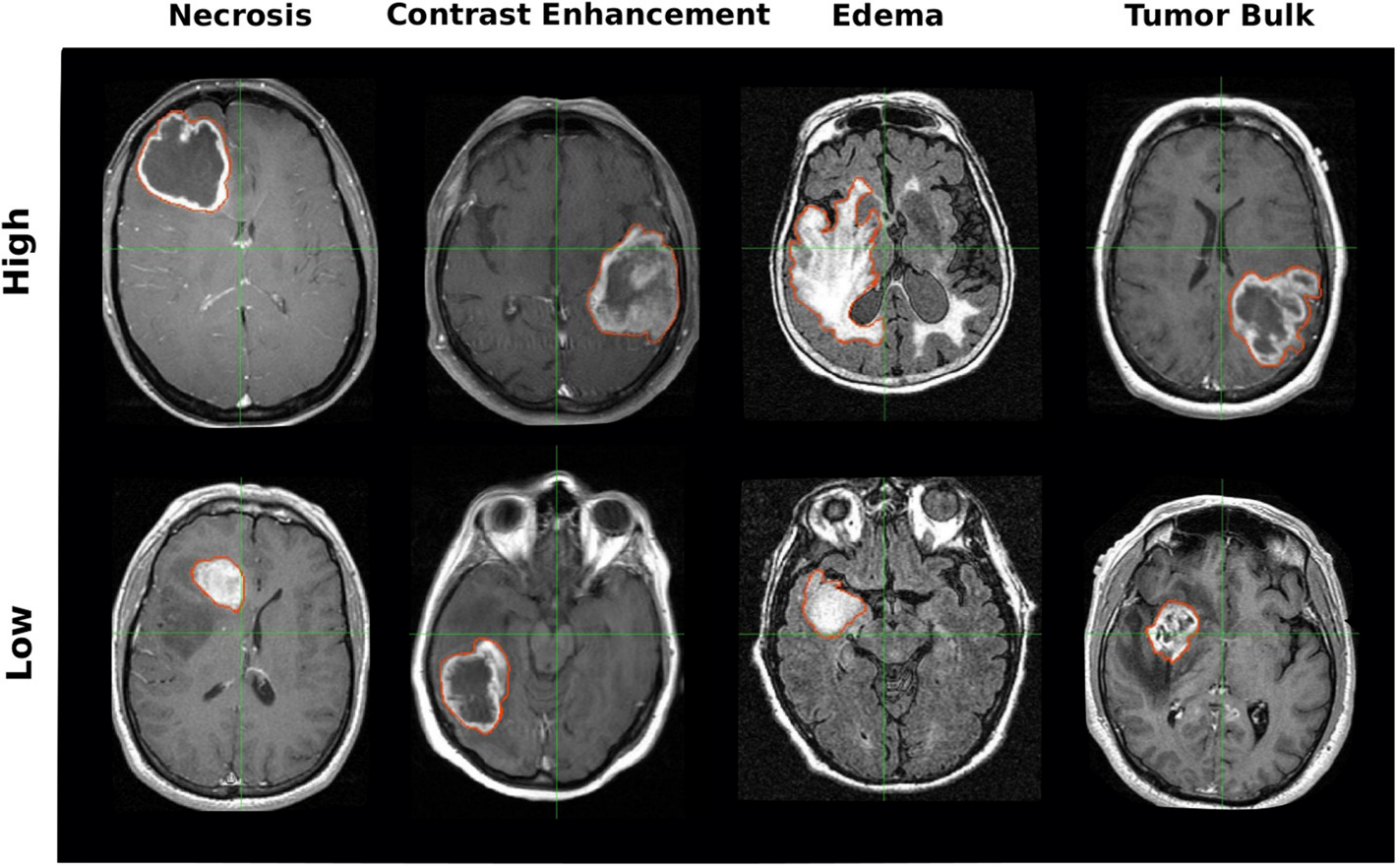
Examples of volumetric tumor phenotype features. Glioblastoma (GBM) tumors show strong phenotypic differences, which can be objectively quantified with volumetrics. This figure shows examples of GBM tumors exhibiting high (top) and low (bottom) volumetric feature values for Necrosis, Contrast Enhancement, Edema, and Tumor Bulk (columns) as they appear on T1 weighted (columns 1,2, and 4) or T2-FLAIR (column 3) magnetic resonance images for different patients

**Fig. 2:**
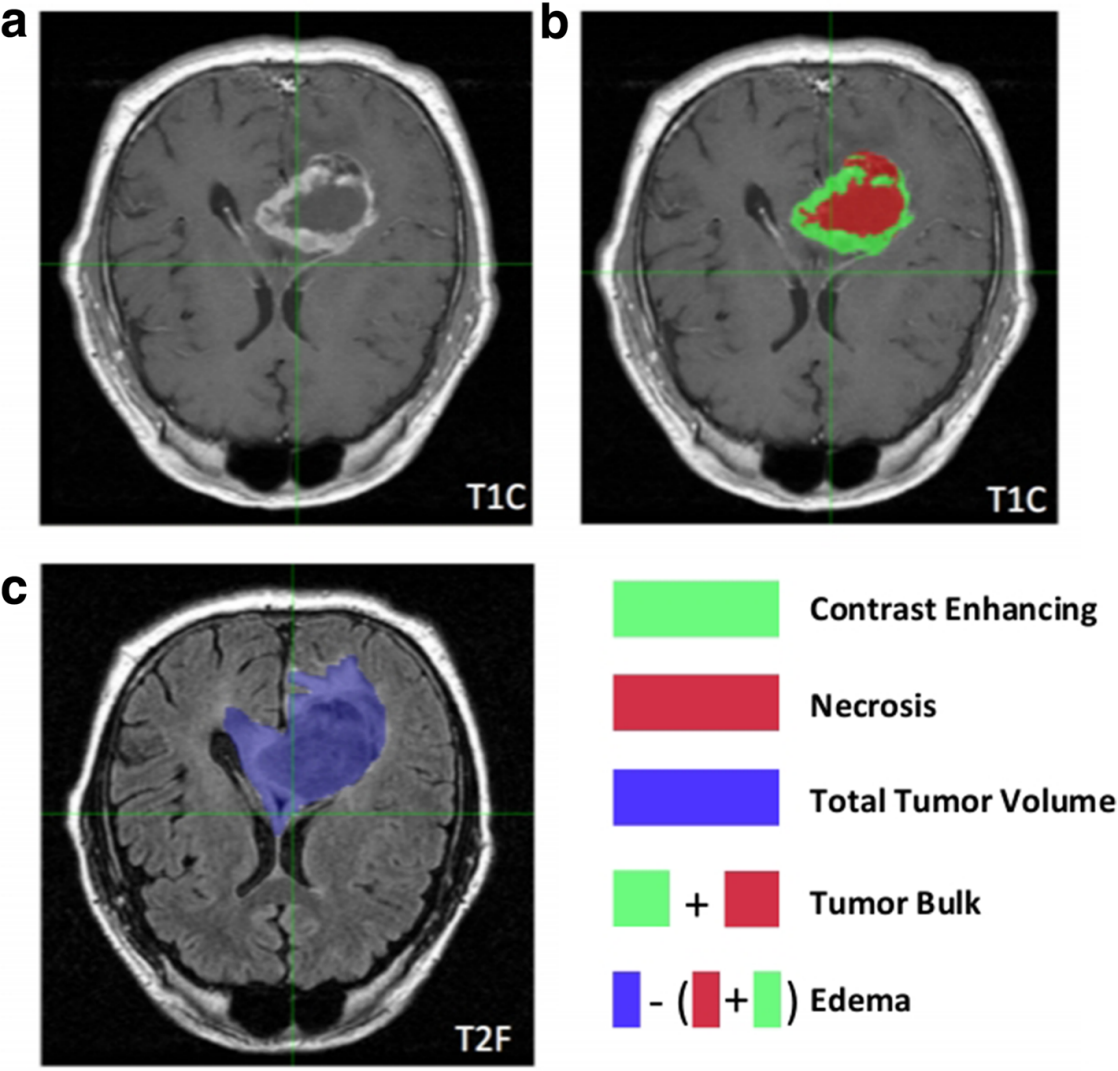
Volumetric phenotype features within the same tumor. Detailed example of a glioblastoma tumor in a patient. (**a**,**b**) On T1-weighted post-Gadolinium contrast (T1C) images, a central area of Necrosis is typically surrounded by a Contrast Enhancing ring, both of which can be derived from dark and light regions, respectively. Tumor Bulk represents the addition of these tumor features. (**c**) The Total Tumor Volume is represented by hyperintensity extracted from T2-FLAIR images. Edema is the difference of Tumor Bulk from Total Tumor Volume

One promising avenue of these investigations involves quantitative analyses of radiographic data, where imaging modalities are used to quantify tumor phenotype noninvasively. In magnetic resonance imaging (MRI), GBM tumors exhibit strong phenotypic features such as Necrosis, Edema, Contrast Enhancement, and Tumor Bulk (Fig. 1). These properties can be captured without and with intravenous administration of gadolinium-based contrast agents including T1-weighted or FLuid-Attenuated Inversion Recovery (FLAIR) (Fig. 2). In this way, visible tumor phenotype features can be systematically quantified.

As the underlying drivers of these phenotypes are biological in nature, recent efforts have been conducted indicating underlying genetic characteristics of imaging features. For example, tumor “Ring Enhancement” was found to be significantly associated with unmethylated MGMT promoter status [4, 5], which is known to be a biomarker for response to temozolomide and survival. Similarly, “Contrast Enhancement” and “Mass Effect” imaging features were found to be strongly correlated with expression of groups of genes involved in hypoxia and proliferation, respectively [6]. However, a systematic classification of tumor phenotype features in terms of their underlying cell biological processes on a genome-wide scale in GBM remains absent, although clinical applicability of these image features will depend on knowledge about how these features are driven by tumor biological processes that determine disease progression.

In this study, we present an Imaging-Genomics analysis to investigate the associations of a large set of biological processes and presurgical diagnostic MRI derived quantitative volumetric tumor phenotype features, such as Necrosis or Edema, focusing on the publicly available GBM dataset from The Cancer Genome Atlas (TCGA). These analyses were tied to molecular subtypes in GBM and prognostics. Image based volumetric features provide noninvasive tumor phenotype information complementary to genomic technologies and clinical information, potentially allowing advances in patient stratification and clinical decision-making.

## Methods

### Magnetic resonance imaging

DICOM formatted files of presurgical T1 and T2 sequence magnetic resonance images (MRIs) were accessed and downloaded in November 2014 from TCIA (https://wiki.cancerimagingarchive.net/display/Public/TCGA-GBM), a large archive of medical images of cancer patients who have matched molecular data at The Cancer Genome Atlas (TCGA). Cases that had both T1 and T2-FLAIR images available, were of reasonable quality to perform tumor segmentation, and had presurgical negative status were included. As the presurgical status of an image is not explicitly included in the TCIA data, presurgical status was verified to the best of our ability by a trained neuroradiologist (CH, 17 years of experience) by examining the skull surrounding the tumor for signs of surgeries. The patients in our study were originally imaged at Thomas Jefferson University Hospital and Henry Ford Hospital.

Images of sufficient quality were next analyzed for volumetric features. Briefly, 2D masks which were annotated using FSLView, a module in the FMRIB Software Library 5.0 (FSL [7]), were applied surrounding the tumor regions on the post gadolinium (GD) contrast T1-weighted images and T2-weighted images. For the T1 images, a single contour was segmented including both the dark (Necrotic or NE) and bright (Contrast Enhancement or CE) areas, and the entire volume was referred to as Tumor Bulk (TB). The pixels contained in these masks were then clustered into dark (NE) and bright (CE) areas by K-means clustering using the FSL FEAT (fMRI Expert Analysis Tool, Version 5.0). The area volume contained within the mask of the T2 FLAIR image set encompasses the Edema (ED) envelope, including regions of hyperintense signal and inclusive of any other abnormal signal in the region previously identified on the T1 (i.e., TB), and was referred to as Total Abnormal Tumor Volume (TV). Afterwards, all masks were visually checked by a trained radiologist (CH). We did not attempt to discriminate between peritumoral edema and non-enhancing tumor, as both appear hyperintense on FLAIR. In addition to the raw volumetric features, we calculated the following feature ratios as investigated in previous studies [8–10] mainly to investigate combined T1/FLAIR signals: NE/TV, CE/TV, ED/TV, TB/TV, NE/CE, and CE/TB. A representation of the tumor volumes analyzed are displayed in Fig. 2.

### Gene expression

Matching GBM gene expression (mRNA) data for the TCIA patient cohort was obtained from TCGA using the CBioPortal [11] with the ‘cgdsr’ R package version 1.1.33. The profile identifier ‘gbm_tcga_pub_mrna_median_Zscores’ was used together with the case identifier ‘gbm_tcga_pub_mrna’ to download the expression values of 18,055 genes given as median Z-scores across the Agilent, Affymetrix U133, and Affymetrix Exon platforms. Expression data were downloaded on April 3, 2015, for 91 patients for which also imaging data was available. Based on expression of 1740 genes, Verhaak et al. [12] classified TCGA-GBM patients into the four molecular GBM subtypes proneural, neural, classical, and mesenchymal, which were functionally annotated by presence of oncogenic events. To test predictive power for subtypes, we downloaded the classification results on TCGA patients by Verhaak et al. from https://tcga-data.nci.nih.gov/docs/publications/gbm_exp/TCGA_unified_CORE_ClaNC840.txt and calculated the multiclass area under curves (AUCs) of the receiver operator characteristic [13] of the volumetric features. Imaging and subtype data were available for 79 patients.

### Pathway analysis

To quantify the association of a volumetric features with biological processes, preranked Gene Set Enrichment Analysis [14] (GSEA) version 2.2.0 was performed; gene ranks were calculated for every feature according to -log10(*p*) *r*, where *r* is the Spearman rank correlation coefficient, and *p* its *p*-value. GSEA was performed on the C5-BP collection version 5.0 from the Molecular Signature Database [15] (MSigDB), which contains the expert-curated Gene Ontology [16] (GO) gene sets for biological processes. Those 583 gene sets containing at least 15 and at most 500 genes were analyzed. We investigated gene sets that were significantly enriched under a false-discovery-rate (FDR) < 0.05 as specified by GSEA to account for multiple hypothesis-testing [17].

### Survival analysis

Overall survival data was available for 141 patients with imaging data, and was downloaded from CBioPortal on April 3, 2015. Prognostic associations of volumetric features were assessed with the concordance index (CI) using the ‘survcomp’ package in Bioconductor [18]. All statistical analyses were carried out using R version 3.1.0 [19] on a Linux operating system.

## Results

To investigate which biological processes drive volumetric tumor phenotype features in GBM, we performed a pathway analysis based on gene expression profiles using a preranked Gene Set Enrichment Analysis [14]. We compared these results to molecular subtypes in GBM and evaluated the prognostic value.

### Volumetric tumor phenotype features in GBM

**Fig. 3:**
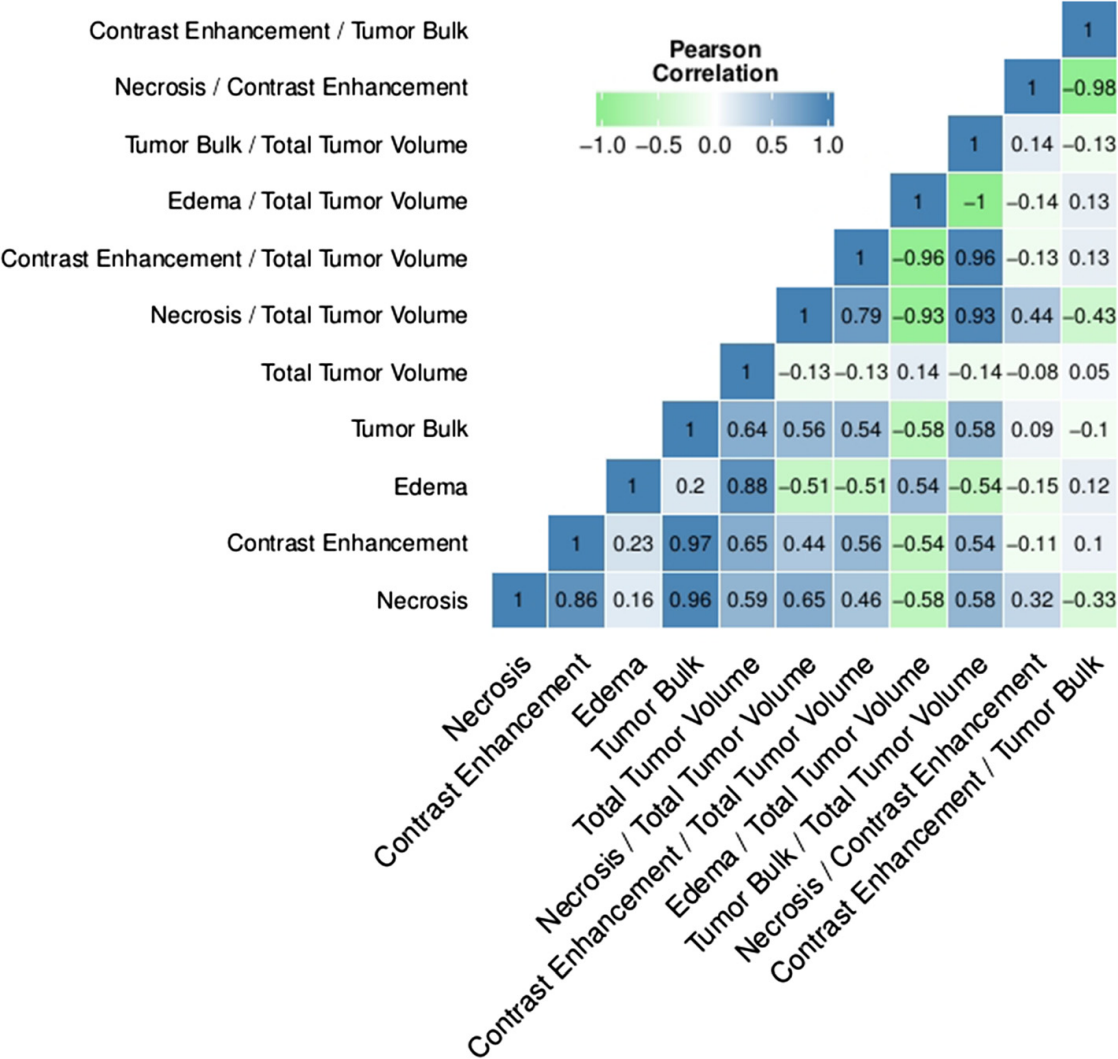
Correlation map. Pairwise Pearson correlation coefficients of volumetric features. Only few volumes were highly correlated (blue) or highly anti-correlated (anti-correlated)

Based on MRI, we quantified the following volumetric features in GBM: Necrosis (NE), Contrast Enhancement (CE), Edema (ED), Tumor Bulk (TB), and Total Tumor Volume (TV). In addition, we calculated the following ratios mainly to investigate combined T1/FLAIR signals: NE/TV, CE/TV, ED/TV, TB/TV, NE/CE, and CE/TB. The areas of the tumor that these features correspond to are highlighted in Fig. 2b and Fig. 2c. In general, we found that these features were not or only moderately correlated (mean Spearman rho 0.48 and -0.41 for positive and negative correlation, respectively), however a number of features were highly positively correlated (e.g., NE and TB, rho = 0.96) and a number of features ratios were highly anti-correlated (e.g., NE/CE and CE/TB, rho = -0.98) as shown in Fig. 3.

### Biological processes underlying volumetric features

**Fig. 4:**
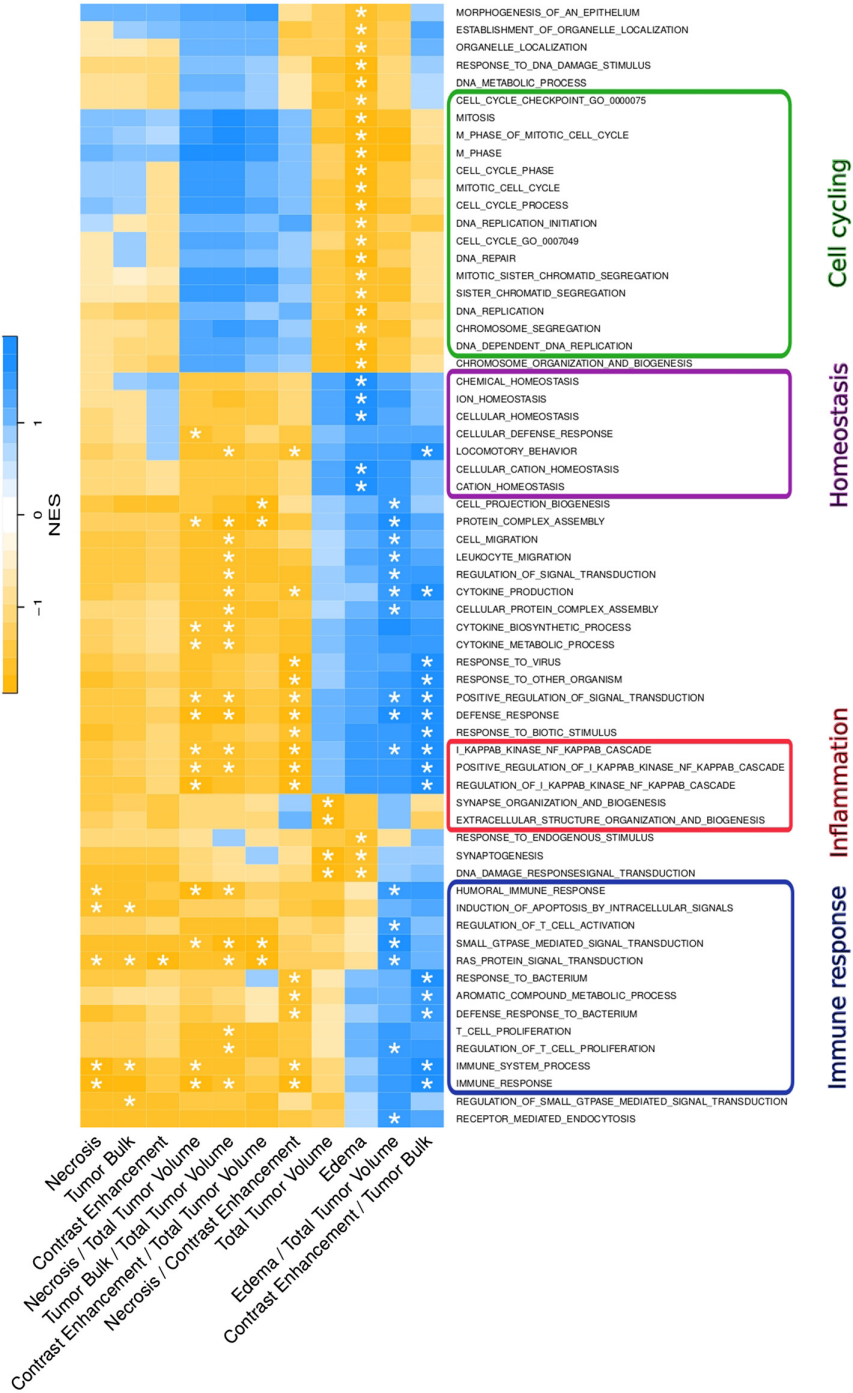
Pathway enrichment analysis. In total, 64 biological processes (rows) were significantly (FDR < 0.05) enriched for at least one volumetric feature (columns) as indicated by an asterisk. Heatmap shows normalized enrichment scores (NES) calculated with Gene Set Enrichment Analysis. Positive NES (*blue*) correspond to correlated pathways and negative NES (*yellow*) correspond to anti-correlated pathways

In total, 64 biological processes were significantly associated in at least one of the volumetric features or their ratios (FDR < 0.05, Fig. 4). Table 1 summarizes the biological themes associated with each volumetric feature. These features were generally negatively (anti-correlated) enriched for biological processes unless stated otherwise. NE and TB were mainly enriched for pathways involved in immune response and apoptosis, whereas CE was enriched for signal transduction and protein folding processes. ED was enriched for cell cycling, proliferation, and replication mechanisms, but also positively enriched for homeostasis. TV was associated with synaptogenesis, biogenesis, and excretion.

**Table 1:** Summary of pathways associated with volumetric tumor phenotype features of the original volumes (top rows) and their ratios (bottom rows)

	Biological processes (positive correlation)	Biological processes (negative correlation)
Volume		
Necrosis		Immune response, apoptosis
Contrast Enhancement		Signal transduction
Edema	Homeostasis	Cell cycle, proliferation, replication, DNA repair, DNA metabolic process
Tumor Bulk		Apoptosis, signal transduction, immune system
Total Volume		Synaptogenesis, biogenesis, extracellular structure organization
Ratios		
Necrosis/Total Volume		Defense response, immune response, Nf-kB, signal transduction
Contrast Enhancement/Tumor Volume		Protein complex assembly, signal transduction, biogenesis
Edema/Tumor Volume	Protein complex assembly, defense response, signal transduction, cytokine production, immune response, Nf-kB	
Tumor Bulk/Tumor Volume		Signal transduction, protein complex assembly, cytokine, immune response, Nf-kB
Necrosis/Contrast Enhancement		Response to other organism, Nf-kB, immune response, locomotory behaviour
Contrast Enhancement/Tumor Bulk	Response to other organism, Nf-kB, immune response, locomotory behaviour	

Volumetric feature ratios were associated with a larger number of biological processes than the original features. Signal transduction was associated with all of the ratios we computed; processes involved in immune system were found for all ratios except for CE/TV. CE/TV, TB/TV, and ED/TV were enriched for protein complex assembly. In addition, ED/TV showed positive enrichment for defense response, cytokine production, and Nf-kB. Nf-kB was also found in NE/TV and TB/TV, as well as in NE/CE and CE/TB. Notably, NE/CE and CE/TB were also inversely enriched for inflammation, immune system response pathways, and anti-apoptosis.

### Molecular subtypes in GBM

**Fig. 5:**
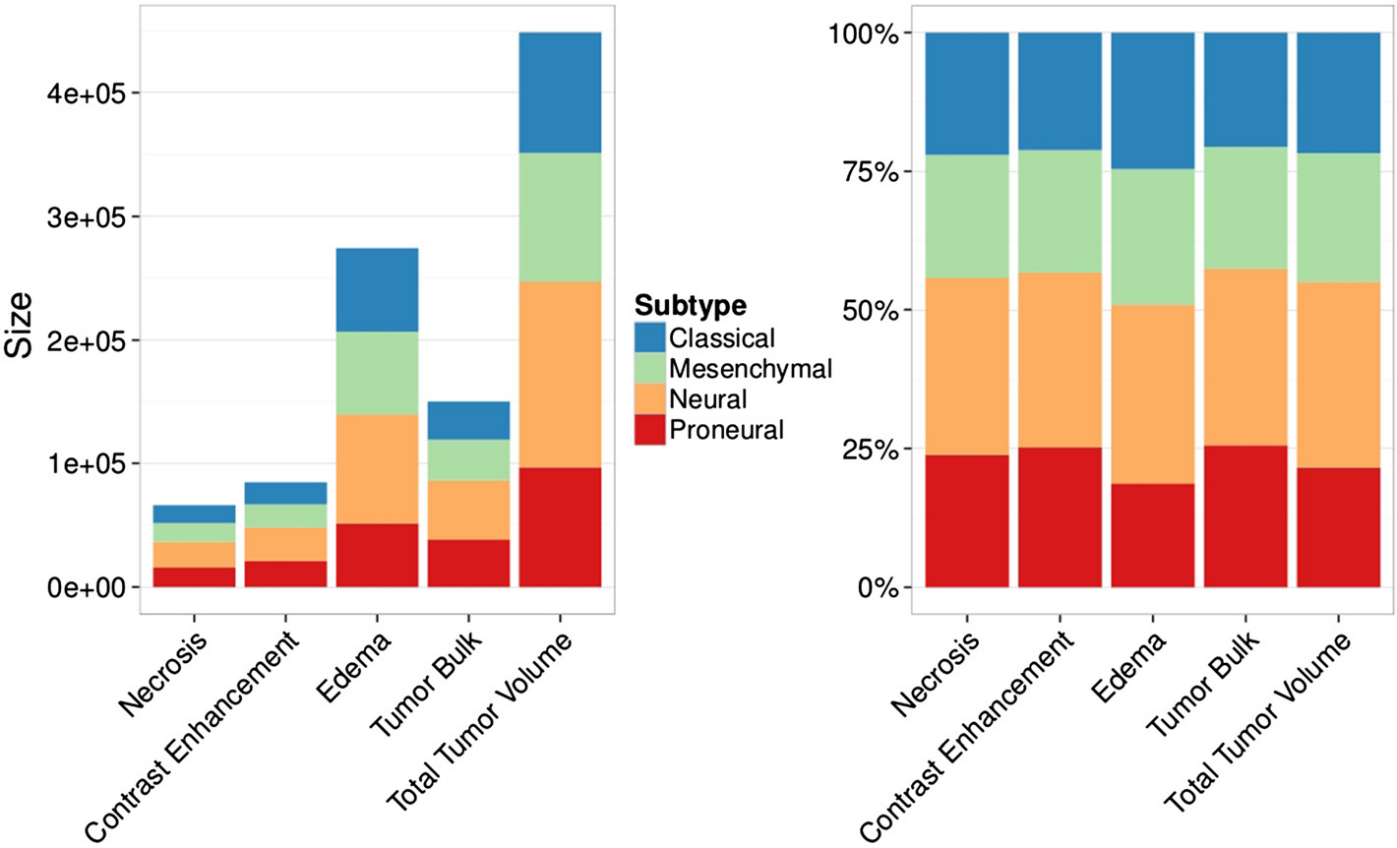
Size distribution of volumetric tumor features across molecular subtypes of GBM. (**a**) Compared to the Total Volume, Edema had the largest median size across all molecular GBM subtypes. (**b**) Classical and neural tumors showed larger Edema areas than mesenchymal and proneural tumors. Size variation of volumetric feature areas other than Edema was generally low across subtypes

Based on a study by Verhaak et al. [12], patients from the TCGA-GBM cohort were classified to belong to either one of the four following molecular subtypes: proneural, neural, classical, and mesenchymal. Compared to TV, ED had the largest median size across subtypes (Fig. 5a); ED was larger in classical GBM and smaller in proneural (Fig. 5b). Other volumetric features were comparably similar in terms of median values across subtypes.

We tested predictive value for GBM subtypes of all volumetric features by calculating the area under the curve (AUC) of the receiver operator characteristic. We found that most features performed relatively low (Table 2). ED and TV were the strongest predictors of subtypes (AUCs = 0.61). Ratios of features generally were poor predictors of subtype.

**Table 2:** Performances of volumetric features in predicting molecular subtypes of GBM

Volume	Multiclass AUC
Necrosis	0.57
Contrast Enhancement	0.57
Edema	0.61
Tumor Bulk	0.57
Total Volume	0.61
Ratios	
Necrosis/Total Volume	0.56
Contrast Enhancement/Tumor Volume	0.55
Edema/Tumor Volume	0.56
Tumor Bulk/Tumor Volume	0.56
Necrosis/Contrast Enhancement	0.54
Contrast Enhancement/Tumor Bulk	0.54

### Prognostic value of volumetrics

**Fig. 6:**
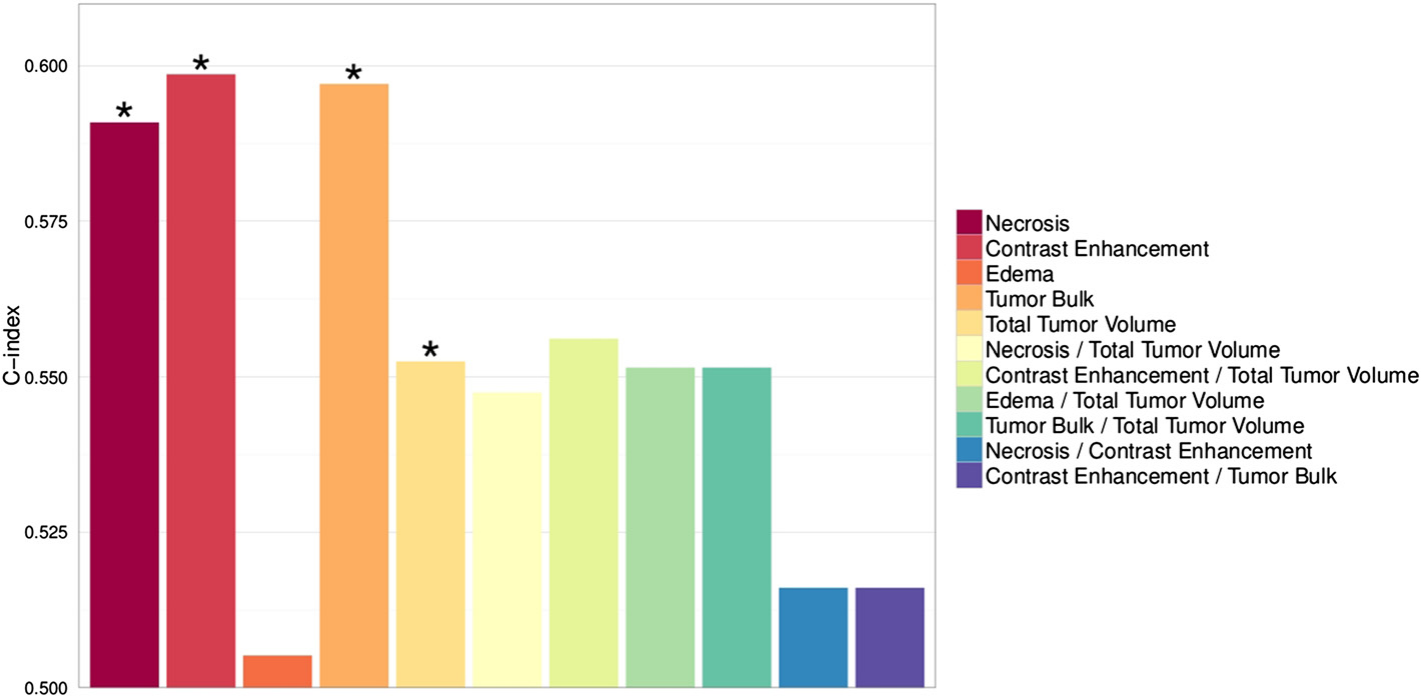
Prognostic value of volumetric tumor features. Necrosis, Contrast Enhancement, Tumor Bulk, and Total Tumor Volume were significantly (asterisk) prognostic (*p* < 0.05). The Contrast Enhancement feature showed the highest prognostic performance as measured by the C-index

To link our pathway-imaging results to clinical patient outcome, we tested prognostic value of volumetric features for overall survival (OS). Four features (NE, CE, TB, and TV) significantly predicted OS (Noether, *p* < 0.05), but prognostic performances as measured by the concordance index [20] were only moderate (Fig. 6). Importantly, NE, CE, and TB performed significantly better than TV (one-sided *t*-test, *p* < 0.05). Furthermore, Kaplan-Meier and Log-Rank analyses revealed significant assessments of low and high risk survival groups by NE, CE, and TB (Additional file 1: Figure S1).

## Discussion

The translation of quantitative imaging data into defined clinical settings requires knowledge of how volumetric tumor phenotype features are driven by biological processes that determine the outcome of a patient. This study presents an Imaging-Genomics analysis of presurgical diagnostic MRI derived volumetric features in GBM to evaluate if tumor phenotype features are associated with underlying tumor biology. We found different features to be enriched for different sets of biological processes. Molecular subtypes of GBM were difficult to be predicted by volumetric features. However, four out of five features showed significant prognostic value.

As correlations among our volumetric features were low to moderate in general, this suggests that quantifying each of those areas individually yields complementary information about the tumor phenotype beyond the Total Tumor Volume (TV). Interestingly, we found most of the biological processes to be anti-correlated to pathway expression. The most prevalent pathways were apoptosis, immune system, and signaling pathways, which were observed mainly for Necrosis (NE), Contrast Enhancement (CE), and Tumor Bulk (TB); features that were also significantly prognostic. As those pathways are known drivers of survival outcome [21–23], this hence explains why NE, CE, and TB were found to be prognostic as well. Importantly, all of these features performed significantly better than TV, which highlights that quantification of individual imaging features should be preferred over calculating only the total tumor volume. Our finding that NE is anti-correlated with immune response and prognostic is in line with Gevaert et al. [24], who also correlated quantitative imaging features of GBM areas to molecular data and who found significant imaging associations to approximately 20 pathways. This analysis, however, differs from our analysis in that Gevaert et al. investigated a single slice of a tumor (in axial view), whereas we performed quantification using the 3D tumor volumes.

Edema (ED) was the only feature that was correlated with homeostasis, cell cycling, and proliferation pathways. Surprisingly, ED was not prognostic in our analysis, although cell cycling and proliferation are known to be involved in carcinogenesis [25]. However, using the publicly available MRI scoring scheme VASARI (https://wiki.nci.nih.gov/display/CIP/VASARI), Gutman et al. [26] found ordinal ED assessment to be not prognostic as well. Interestingly, in a related study by Diehn et al. [6], binary assessment of ED resulted in significant survival predictions. Similarly, a recent study indicated that an ED volume cutoff of 85’000 mm^3^ is a significant prognostic factor using Kaplan-Meier analysis [8]; however, the rationale for this cutoff was not given. Prognostic performance of quantitative ED features could increase in cohorts of extended sample sizes, as ED has been reported to be a univariate predictor of survival in a large patient cohort previously [27]. Furthermore, our analysis suggests that CE and NE are prognostic. This is partially in line with Gutman et al. [26], according to whom CE is prognostic, but NE is not. The contradictions between our results and the studies by Diehn et al. and Gutman et al. could be due to the nature of the ED and NE assessments, which in our analysis were continuous, but binary and ordinal in Diehn et al. and Gutman et al., respectively. Likewise, our methodology could be compared to a study by Jamshidi et al. [28], but comparison remains challenging as their analysis focused on binary imaging traits on a relatively small cohort of patients and a subset of oncogenic pathways only.

Although ED was the only feature that was not prognostic in our analysis, it was the highest predictor of molecular subtypes in GBM instead. This is likely because ED was the only feature that expressed a different volumetric size distribution across molecular subtypes. Similar indications have been given by Gevaert et al. [24], who found three out of four features that correlated with molecular subtypes to be quantitative Edema features. In general, we found volumetric features to be only moderate predictors of subtypes suggesting that subtypes do not generally alter the size composition of tumor areas in GBM. Furthermore, we could not confirm that the proneural subtype has lower proportions of CE as suggested by Gutman et al. [26]. Poor predictability of Verhaak molecular subtypes by relative cerebral blood volume using T2-weighted MRI has been also described by Jain et al. [29].

In our analysis, ratios of volumetric features were not significantly prognostic or predictive of GBM subtypes. Generally, many more biological processes were significantly associated with the feature ratios, usually showing a trend towards a mix of pathways associated with the individual features that the ratios were composed of (e.g., NE/TV were enriched for signal transduction and biogenesis). While our study associated MRI volumetric features with biological processes, molecular subtypes, and survival outcome using genome-wide data and aimed at explaining the rationale for why MRI derived volumetric features are associated with survival on a pathway level, other studies have focused on revealing specific genetic variations between MRI features and survival [30–34].

Our analysis was limited to a retrospective dataset. To establish volumetric biomarkers in clinical applications, prospective evaluation of our results will be required. Biological significance could be further validated by analyses of complementary molecular data such as mutational or epigenetic data. Such analyses could provide further insight into why separate quantification of distinct volumetric tumor phenotype features yield different biological and prognostic information. We acknowledge that the prognostic and predictive performances of the volumetric features in the TCGA-GBM dataset were moderate. Heterogeneity of GBM tumors [35] could be an explanation for this, which limits the definition of a single molecular subtype especially on the basis of single-needle biopsy [36, 37]. As imaging approaches target the entire visible tumor, we however expect in future studies that prognostic performances will drastically increase when Imaging-Genomic cohorts with even larger numbers of samples and standardized image processing become available for GBM.

While our study focused on volumetric phenotype features, alternative definitions of imaging phenotypes are available. This may, for example, include tumor location as this determines the extent of possible resection and hence is a prognostic factor in GBM [38]. In addition to such semantic phenotypes, agnostic phenotyping approaches such as radiomics could be added [39].

## Conclusions

In conclusion, quantitative imaging biomarkers hold great potential, as, unlike traditional biopsies, medical imaging is noninvasive and captures the entire tumor volume. As we have shown, a relationship exists between individual volumetric phenotype features describing local, clinically relevant subareas of GBM tumors and global expression of genes. Knowledge about how these specific tumor areas are related to underlying biological cell processes may allow for advanced patient stratification and treatment decision on the basis of standard medical imaging, but efforts in optimization of prognostic and predictive performances need to continue.

## Additional file

Additional file 1: Figure S1. Stratification power of volumetric tumor phenotype features. Kaplan-Meier analysis of the volumetric features that showed significant prognostic value (i.e., Necrosis, Contrast Enhancement, Tumor Bulk, and Total Tumor Volume). Except for Total Tumor Volume, these features also showed significant classification in low (blue) and high (red) risk groups based on the mean feature value. (PDF 111 kb)
