# Supplementary figures and images for: Imaging-genomics reveals driving pathways of MRI derived volumetric tumor phenotype features in Glioblastoma
Source: BMC Cancer. 2016 Aug 8;16:611. doi: 10.1186/s12885-016-2659-5 (PMC4977720; doi:10.1186/s12885-016-2659-5)

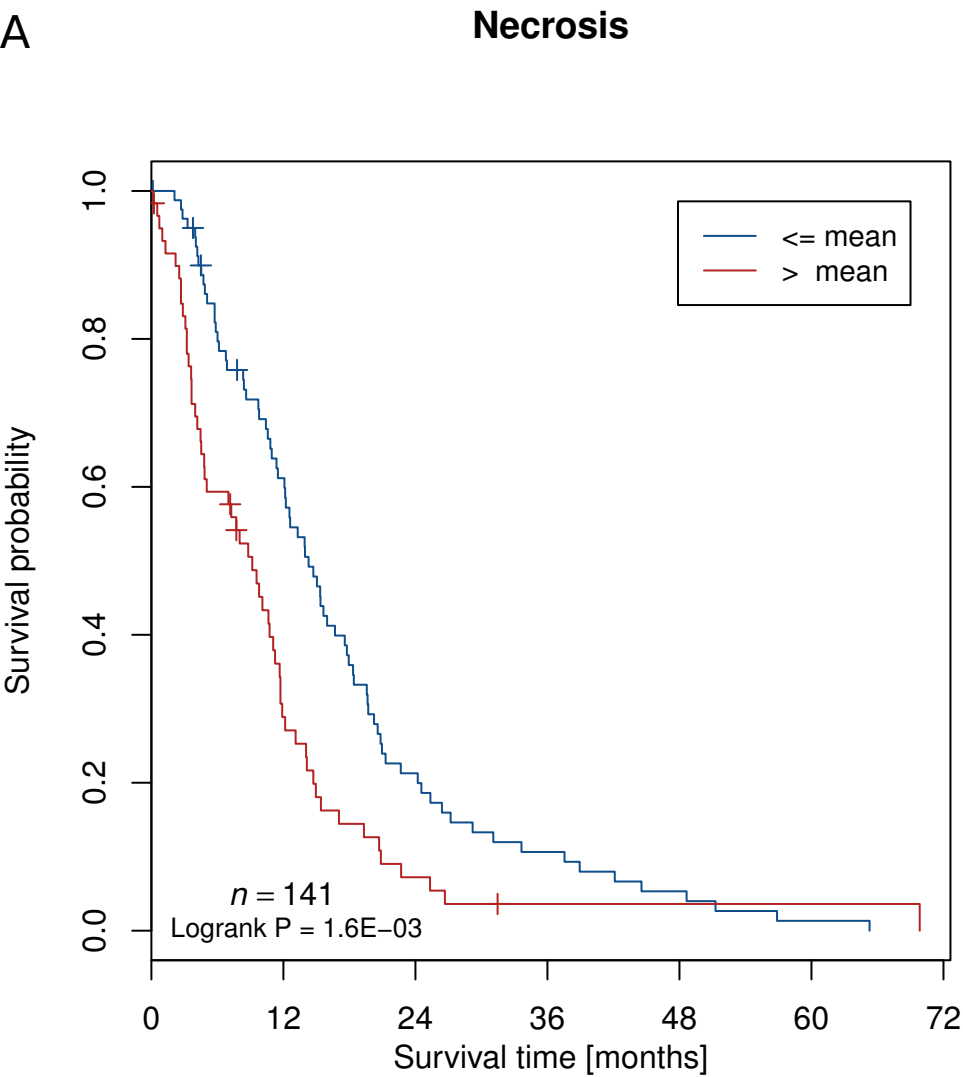

**No. At Risk**

|         |    |    |    |   |   |   |   |
|---------|----|----|----|---|---|---|---|
| <= mean | 81 | 46 | 16 | 8 | 4 | 1 | 0 |
| > mean  | 60 | 16 | 4  | 1 | 1 | 1 | 0 |

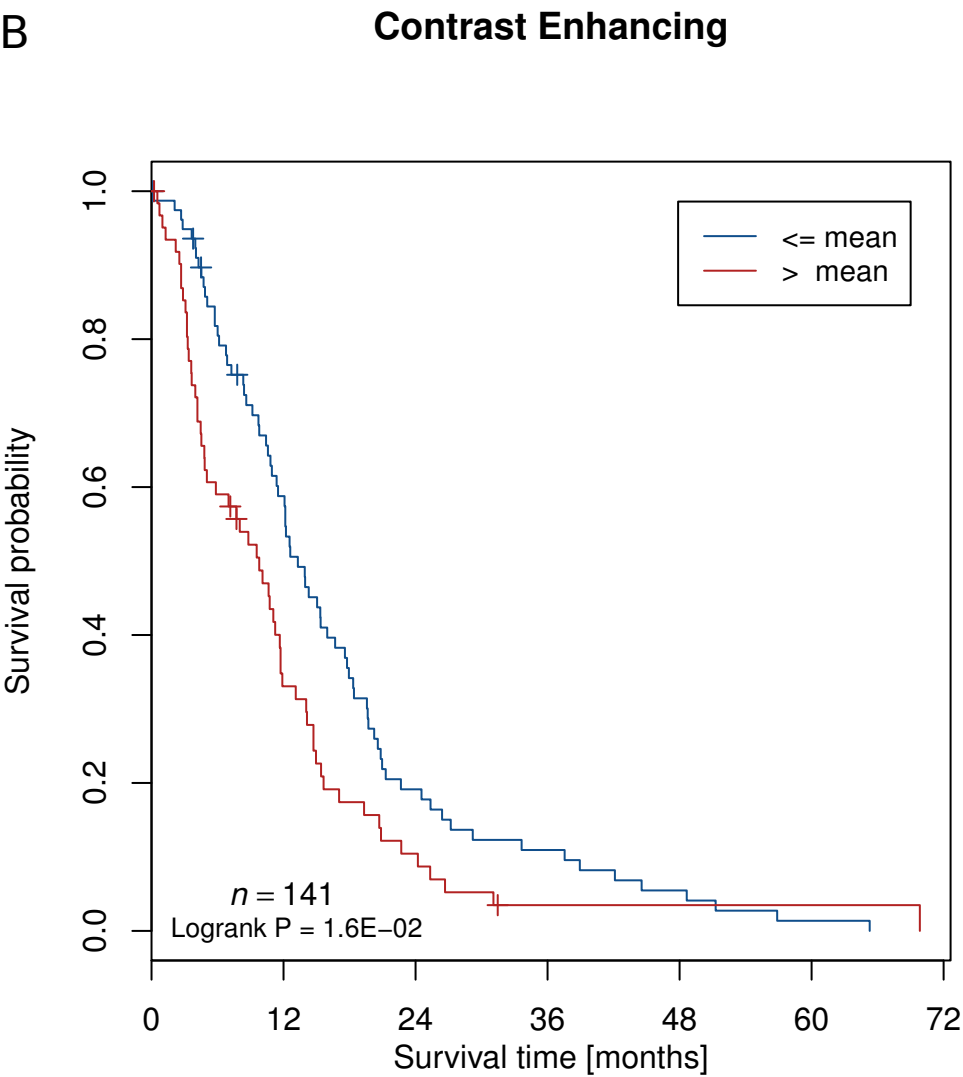

**No. At Risk**

|         |    |    |    |   |   |   |   |
|---------|----|----|----|---|---|---|---|
| <= mean | 79 | 43 | 14 | 8 | 4 | 1 | 0 |
| > mean  | 62 | 19 | 6  | 1 | 1 | 1 | 0 |

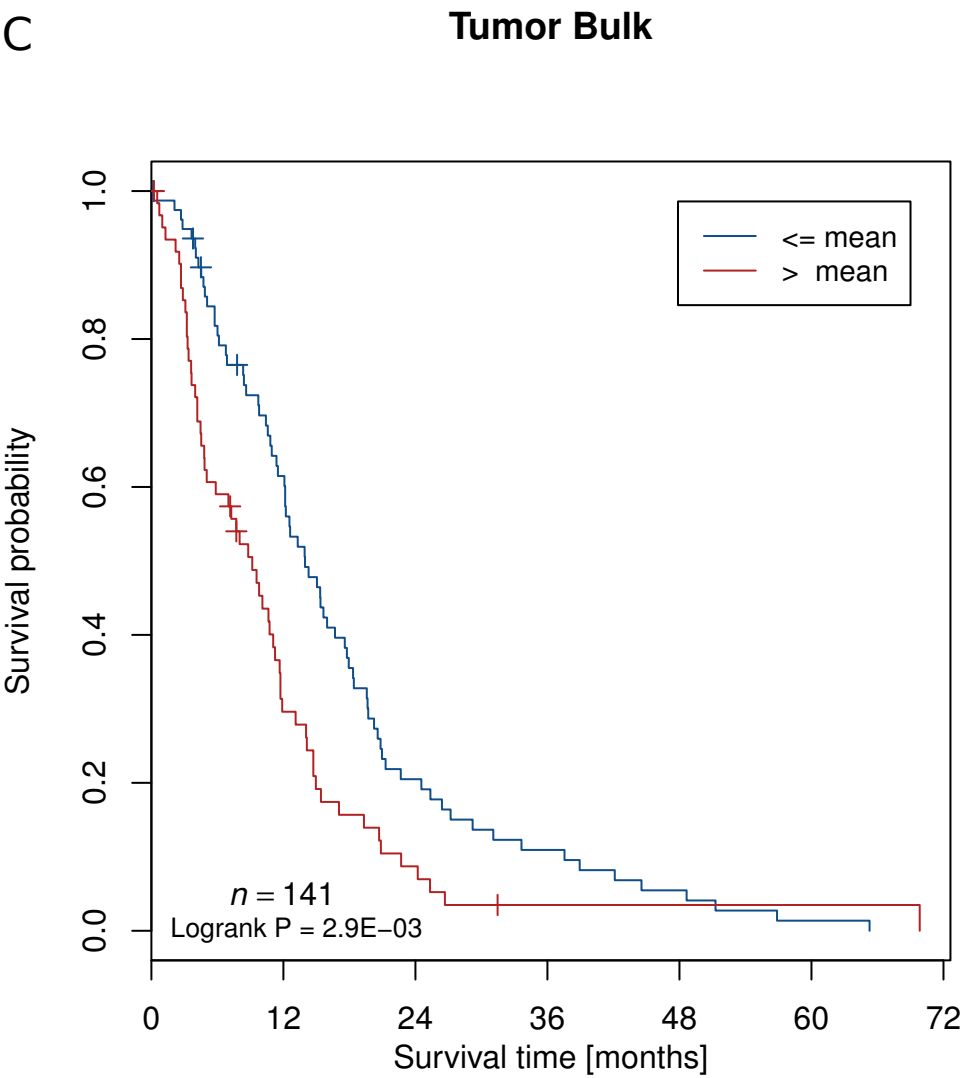

**No. At Risk**

|         |    |    |    |   |   |   |   |
|---------|----|----|----|---|---|---|---|
| <= mean | 79 | 45 | 15 | 8 | 4 | 1 | 0 |
| > mean  | 62 | 17 | 5  | 1 | 1 | 1 | 0 |

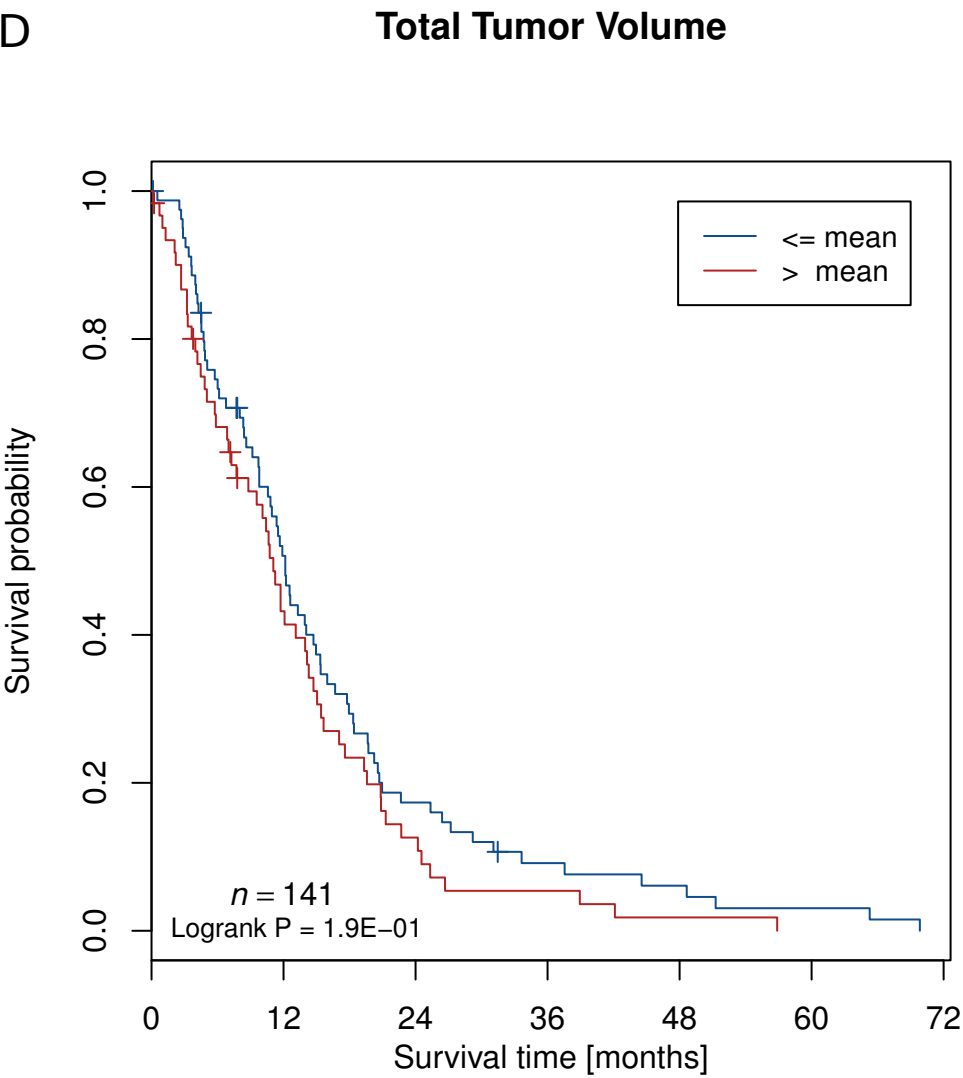

**No. At Risk**

|         |    |    |    |   |   |   |   |
|---------|----|----|----|---|---|---|---|
| <= mean | 80 | 38 | 13 | 6 | 4 | 2 | 0 |
| > mean  | 61 | 24 | 7  | 3 | 1 | 0 | 0 |

Supplement: Additional file 1: Figure S1. — Stratification power of volumetric tumor phenotype features. Kaplan-Meier analysis of the volumetric features that showed significant prognostic value (i.e., Necrosis, Contrast Enhancement, Tumor Bulk, and Total Tumor Volume). Except for Total Tumor Volume, these features also showed significant classification in low (blue) and high (red) risk groups based on the mean feature value. (PDF 111 kb) [file 12885_2016_2659_MOESM1_ESM.pdf]
